# Cognitive-Motor Dual Task Interference Effects on Declarative Memory: A Theory-Based Review

**DOI:** 10.3389/fpsyg.2020.01015

**Published:** 2020-05-26

**Authors:** Phillip D. Tomporowski, Ahmed S. Qazi

**Affiliations:** Department of Kinesiology, University of Georgia, Athens, GA, United States

**Keywords:** executive function, embodied learning, long-term memory, attentional allocation, memory consolidation, physical activity, arousal theory

## Abstract

Bouts of exercise performed either prior to or immediately following study periods enhance encoding and learning. Empirical evidence supporting the benefits of interventions that simultaneously pair physical activity with material to be learned is not conclusive, however. A narrative, theory-based review of dual-task experiments evaluated studies in terms of arousal theories, attention theories, cognitive-energetic theories, and entrainment theories. The pattern of the results of these studies suggests that cognitive-motor interference can either impair or enhance memory of semantic information and the manner in which physical activity impacts working memory within executive processing appears to explain disparate outcomes. The integration and timing of physical movements in concert with the type of information to be encoded and remembered appears to be a critical requirement for learning. These observations have implications for the role of physical activity in education, rehabilitation, and gerontological settings.

## Introduction

Routine, long-term chronic exercise is known to alter physical and mental functions. There is considerable evidence that exercise training benefits brain health and promotes improvements in information processing speed, executive function, and attention (Erickson et al., 2015). Historically, these augmentations have been explained in terms of adaptation of neurological structures and processes. There is compelling evidence for causal relations between exercise training and changes in brain structures and neurotrophic factors (Hillman et al., 2017). These findings have led researchers to consider a dose-response relation between exercise and mental function. Recent emphasis has been placed on psychological processes that may play a role in explaining the exercise-cognition relation. Reviews of the exercise literature reveal the importance of exercise interventions that engender goal-setting, mental engagement, and personal relevance (Diamond and Ling, 2016, 2020; Vazou et al., 2016). An examination of chronic exercise training experiments that made direct comparisons between interventions that emphasized routine aerobic or strength-training routines and multicomponent interventions that combined exercise training with cognitive training suggested that there are added benefits from multicomponent interventions (Tomporowski and Pesce, 2019), explaining the added benefit in terms of the presence of dual-task demands that optimize physical and mental challenge. Hypotheses were made concerning the importance of instructional methods and the necessity for varying the manner in which individuals process information, make decisions, select movements, and experience the consequences of their actions.

The mental operations that underlie dual- and multicomponent task performance have been studied extensively and several theories have been proposed to explain how individuals optimize decision making when placed in conditions in which two or more tasks are performed simultaneously (see historical review Wickens, 2008). In these theories, the roles of executive function (e.g., working memory, switching, and inhibition) are central to explaining how people deal with cognitive workload demands. In the main, theorists tend to view executive function in terms of “cool” information processing; that is, decision making and learning based on the manipulation of abstract concepts, number, or letters (Hongwanishkul et al., 2005; Brock et al., 2009). Less often is executive function described in terms of “hot” information processing, which refers to the affective responses that are evoked by motivationally and emotionally meaningful instructional contexts (Kerr and Zelazo, 2004; Zelazo and Kesek, 2010). This distinction is particularly relevant when considering conditions designed to pair emotionally laden physical activities with cognitive tasks. For example, exergames that couple exercise such as ergometer cycling with memorization of foreign language words embedded in video-scenes (see review Stojan and Voelcker-Rehage, 2019). Under these conditions, cognitive-motor interference (CMI) may occur. A task-classification system developed by Plummer et al. (2013) describes nine dual-task conditions and their possible outcomes: “(1) no interference (performance of either task does not change relative to single-task performance); (2) cognitive-related motor interference (cognitive performance remains stable while motor performance deteriorates); (3) motor-related cognitive interference (motor performance remains stable while cognitive performance deteriorates); (4) motor facilitation (cognitive performance remains stable while motor performance improves); (5) cognitive facilitation (motor performance remains stable while cognitive performance improves); (6) cognitive priority trade-off (cognitive performance improves while motor performance deteriorates); (7) motor-priority trade-off (motor performance improves while cognitive performance deteriorates); (8) mutual interference (performance of both tasks deteriorates); or (9) mutual facilitation (performance of both tasks improves). The pattern of CMI likely depends on several factors, including the types of tasks; levels of difficulty; instructions regarding which, if any, task to prioritize; and the characteristics of the person performing the task (e.g., cognitive and motor abilities, fear of failing, and distractibility).”

Central to the present review is the influence of the type of physical activity that is paired with learning semantic memory tasks. Exercise-induced arousal has long been considered a vehicle to boost attention and learning. Individuals who intentionally cycle on ergometers or walk on treadmills while reading, listening to podcasts, or viewing language instruction videos often believe that the material will be retained better when accompanied with physical activity. Indeed, a commercial industry has emerged that promotes the benefits of exergaming, which pairs exercise with learning tasks. As a result, some educators have considered the value of embedding in-class physical activities into their academic curricula (Mavilidi et al., 2018b). Given the multiple outcomes associated with CMI, it is deemed important to examine studies that have been conducted that pair physical activity with the encoding of semantic information.

The impact of individual bouts of exercise on cognition has been studied extensively and numerous reviews and meta-analyses consistently report temporary improvements in executive function and memory (Chang et al., 2012; Hillman et al., 2019; Lambourne and Tomporowski, 2010). These reviews, however, evaluate studies designed to evaluate how a bout of exercise impacts later cognitive processing. Lacking is information concerning conditions in which physical movement and cognitive processing occur simultaneously and how these dual-task conditions influence executive functions and affect the strength of memory encoding. A close inspection of the separate and interactive roles of hot and cool forms of executive function may elucidate how bouts of exercise facilitate memory storage. Presently, consolidation theory is the leading explanation for the transfer of information from short-term to long-term memory. Numerous laboratory studies highlight the role stressors play on the activation of the amygdala and the initiation of molecular time-locked processes involved in long-term potentiation that occur at the neuronal level that promote memory storage (McGaugh, 2015; Roig et al., 2016; Loprinzi et al., 2017). Studies conducted primarily with animals show that environmental stressors such as electrical shock and stimulant drugs enhance memory for events preceding stressors (McGaugh, 2000). Of note, proponents of cool and hot executive function make a distinction between the impact of environmental stressors that elicit reflexive stress responses and bottom-up sensory inputs to the amygdala (which negatively impact executive function) and learning tasks that create a context of challenge and problem-solving that activates orbitofrontal cortex and other medial brain regions (Zelazo and Carlson, 2012). While exercise does stress the systems of the body, the pattern of activity differs from those elicited by threating environmental stressors that elicit widespread affective response patterns (i.e., startle, freezing, piloerection, and facial expressions of fear; Dishman and Jackson, 2000). Thus, bouts of exercise that provide a context for skill development may lend themselves toward positive affective experiences, engender goal-directed motivated action plans, and maintain mental engagement.

The cognitive benefits derived from long-term chronic exercise training emerge from repeated, individual exercise bouts. As such, a close examination of dual-task studies that combine exercise in a single session or over a limited number of sessions with the encoding and retention of semantic information, may provide insight as to why long-term chronic experiments have shown added benefits from multicomponent interventions. Of particular interest is the identification of specific methodological factors that either may facilitate or compromise long-term memory. Interpretation of these factors could serve to bolster contemporary theory development, as well as to enhance clinical applications of basic science to targeted populations.

## Methods

Considerable research has examined task interference that results from attempts to deal with two or more cognitive tasks (e.g., performing a mathematics problem while listening to an ongoing conversation). The focus of the present review is on experiments in which dual-task conditions include a cognitive task (i.e., memorization) while engaged in physical activity (e.g., exercise). Sixteen experiments were retrieved following topical key-term searches. Search terms included: Acute exercise AND perceptual memory, episodic memory, associative memory, instrumental learning, relational learning, consolidation, procedural learning, implicit learning, action-perception learning, embodied learning, declarative memory, sematic memory, spatial memory, emotional memory, entrainment memory, sensory memory, iconic memory, echoic memory, imagery, dual-coding theory, eidetic memory, long-term potentiation, prospective memory, and meta-memory. Sources of studies published between 1985 and 2019 included Pubmed, Scopus, EBSCO, Google Scholar, and personal literature bases. Studies selected were restricted to those employing bouts of exercise or physical activities that require controlled actions of major muscle groups and induce a physical workload (e.g., walking, running, classroom activities, and sport skills) while encoding words into long-term memory. Study designs included single-trial and brief multi-trial experiments. Experiments evaluating balance and fine psychomotor movements were excluded. The purpose of the present selective review is to evaluate examples of dual-task exercise interventions that are representative of specific theories of long-term memory. Our intent was not to conduct a systematic review or a meta-analysis but rather to focus on methods employed in interventions designed to influence long-term memory and learning.

## Results

A cursory evaluation of the methods and outcomes in these experiments revealed considerable variation, with several studies demonstrating dual-task costs when exercise was performed simultaneously with encoding and other studies providing evidence of long-term memory enhancement (see Table 1). In an attempt to reconcile the different outcomes observed among these studies, the theoretical assumptions that guided the protocols used in individual experiments were evaluated and study outcomes were appraised. Experiments that have examined dual-task conditions in which exercise is performed simultaneously with the encoding of semantic information have been conducted to test arousal theory, attention theory, cognitive-energetic theory, and entrainment theory. It is recognized that there is considerable overlap among these theories. Nevertheless, each theory emphasizes a specific theme, or construct that guides the methods and tests that are employed by researchers. The main characteristics of each of these theories are provided in Table 2.

**TABLE 1:** Selected studies that assess the effects of cognitive-motor interference on long-term memory.

**Authors**	**Age (years)**	**Number**	**Intervention type**	**Memory task & test**	**Retention interval**	**Comparison conditions**	**Results**
**Arousal and Neuropsychological Theories**							
Soga et al., 2017	22	20	Cycling (12 min) Moderate intensity 60–70% HR_max_	Visual source memory task Retrieval	5 min	Within subject	DTCs^1^ lower item recognition accuracy
Frith et al., 2017	22	88	Treadmill running (15 min) Vigorous (RPE 16-20)	Auditory Verbal Learning Task Prospective Memory	20 min 24 h	Control Pre-^3^ encoding Post-^4^ encoding	20 min: Free call Pre^3^ > SIM/ Control 24 h: Recognition Pre > SIM/ After
Sng et al., 2018	23	88	Treadmill walking (15 min) Preferred pace Moderate intensity 58% HR_max_	Auditory Verbal Learning Test Prospective memory	20 min 24 h	Control Pre- encoding Post- encoding	20 min: Free recall Pre > SIM & Post Control > Post 24 h: Recognition Pre > SIM & Post Attribution Pre > Post Control > Post
Tomporowski et al., 2017	22	24	Isometric hand grip (100 s) 50%_max_/10%_max_	Visual word list Free recall Recognition	Immediate Delayed (5 min)	Within subject	DTCs during encoding: Lower word recall & recognition
**Attention Theories**							
Lindenberger et al., 2000	60–70 40–50 20–30	47 45 48	Walking (170 s) (Simple/Complex)	Auditory word list Serial recall	10 s	Standing Sitting	DTCs increased with age DTCs in young
Li et al., 2001	60–75 20–30	40 37	Walking (Adapted speed/ Complexity)	Auditory word list Serial recall	10-s	Single task	DTCs increased with age
Epling et al., 2016	28	12	Vigorous running (5 min) (Adapted speed)	Auditory word list Free recall	Immediate	Single task	DTCs for words
Green and Helton, 2011	22	12	Traverse wall climbing (3 min)	Auditory word list Free recall	Immediate	Single task (Seated)	DTCs for words
Darling and Helton, 2014	27	12	Traverse wall climb (5 min)	Auditory word list Free recall	Immediate	Single task (Seated)	DTCs for words
**Cognitive Energetic Theories**							
Toumpaniari et al., 2015	4–5	67	Animal imitation Gesture Verbal repetition	Visual word-pair list Cued recall	4 weeks	Seated	SIM^2^ > Gesture > Seated
Mavilidi et al., 2016	4–5	87	Animal imitation movement to spatial locations (30 min) Running (30 min)	Object name-location paired association Name- location recall	Immediate 5 weeks	Seated observation	Immediate: SIM = Run > Seated Delayed: SIM = Run > Seated
Mavilidi et al., 2017	4–5	86	Running to spatial locations (10 min) Running (undirected) (10 min)	Object name-location paired association Name-location recall Recognition	Immediate 6 weeks	Seated observation	Immediate + delayed: SIM > Run > Seated
Mavilidi et al., 2018a	4–5	120	Running, jumping, skipping along spatial number line (15 min) Running (15 min)	Counting Number line estimation Block counting Numerical magnitude comparison Numerical identification	Immediate 6 weeks	Seated observation Control	Immediate + delayed: IPA^5^ > NIPA^6^
Schmidt et al., 2019	8–10	104	Animal imitation Verbal repetition	Auditory-Visual word-pair list Cued recall	Immediate	Seated	IPA^5^ & NIPA^6^ > Seated
**Entrainment & Embodied Learning Theories**							
Schmidt-Kassow et al., 2013	22	81	Cycling (30 min) 69% HR_max_	Auditory word list Cued recall	48 h	Seated rest Pre exercise	SIM > Rest/Pre
Schmidt-Kassow et al., 2014, Exp #1 Exp #2	22 22	18 31	Treadmill (30 min) Light intensity Treadmill (30 min) Light intensity	Auditory word list Cued recall Auditory word list Cued recall	24 h 24 h	Within subject Within subject	SIM > Rest SIM > Rest

*DTCs^1^ = Dual-Task Costs; SIM^2^ = Simultaneous; Pre^3^ = Exercise prior encoding; Post^4^ = Exercise after encoding; IPA^5^ = Integrated physical activity; and NIPA^6^ = Nonintegrated physical activity.*

**TABLE 2:** Central assumptions of theories prompting dual-task physical activity research.

**Arousal Theories** Assumptions: Metabolic activity increases with physical activity and results in alterations in brain neuromodulation. Hypotheses: Exercise-induced arousal affects cognition in an inverted-U fashion and mental performance is enhanced with moderate, but not high, levels of arousal.
**Attention Theories** Assumptions: Attention serves to “gate” what sensory experience enter consciousness and how it is manipulated in working memory. Hypotheses: Interference among items held in short-term working memory compromises transfer into long-term memory.
**Cognitive-energetic Theories** Assumptions: There is an interrelation between top-down cognitive control and bottom up sensory input and processing. Hypotheses: Mental resources are allocated in ways that optimize behavioral actions and their consequences.
**Entrainment Theories** Assumptions: Learning is grounded in physical enactment as opposed to information processing. Hypotheses: The temporal structure of bodily movements organizes sensory input, motor output, and learning.

### Arousal and Neuropsychological Theories

Increases in physical activity lead to a cascade of metabolic responses that signal brainstem nuclei central to the activation of noradrenergic and serotonergic systems that modulate brain activity, particularly in the prefrontal, and parietal cortices (Robbins, 1997; McMorris, 2016). Increased arousal is purported to enhance cognitive processing by altering the signal-to-noise ratio of neurological systems which, in turn, enhances attention, stimulus selection, and decision making (McMorris and Hale, 2015). The facilitative effects of individual bouts of exercise on information-processing speed have been explained in terms of the changes in central nervous system neurotransmitter systems that modulate brain activity (McMorris and Hale, 2015). The relation between arousal and cognitive function has been described in terms of an inverted-U function in which mental performance is enhanced with moderate, but not high, levels of arousal (McMorris and Graydon, 2000; McMorris and Hale, 2015). Heavy exercise elicits a broad spectrum of metabolic changes, peripheral and central fatigue, impaired motor control, and psychophysical perception alteration.

Evidence obtained from neuropsychology research and theory has been drawn upon to explain the relation between acute exercise and long-term memory storage. Comprehensive reviews of potential mechanisms have focused on the impact of physiological arousal prior to and following encoding periods (Roig et al., 2013; McGaugh, 2015; Loprinzi et al., 2017). Following contemporary neuro-biological descriptions of the brain substrates that underlie the formation and storage of memory engrams (e.g., hippocampus, amygdala, and cerebral cortices), researchers have attempted to link acute exercise to specific brain structures and functions (Loprinzi et al., 2017; Hillman et al., 2019). Of particular interest to the present review is the impact of arousal occurring during encoding processes and whether the after-effects of arousal influence memory consolidation.

#### Arousal Research: Experimental Evidence

Several experiments have focused on individual exercise bouts performed at intensities that promote aerobic metabolic energy production. A study conducted by Soga et al. (2017) had young adults perform an ergometer cycling protocol during which individual participants pedaled for 5 min. The workload was then gradually increased to a target heart rate (60–70% HR maximum), which is considered moderate intensity aerobic exercise. Cycling continued throughout a 7-min encoding phase during which participants performed a source memory task that involved viewing 160 pictures composed of 80 objects and 80 animals. Following a 5-min break from cycling, the participant completed a recognition task that included the 160 pictures viewed and 80 new pictures. The participant judged whether the item was old or new (familiarity judgement) and, if old, whether it was an object or animal categorization (source judgement). Comparison of encoding under exercise and non-exercise conditions revealed that simultaneous exercise led to poorer source judgement; there were no differences on familiarity judgements. These behavioral results, together with neuro-electric brain recordings, led Soga and colleagues to conclude that acute aerobic exercise compromised hippocampal processes involved in source judgment but not familiarity judgments, which rely less on hippocampal activation.

A series of experiments conducted by Lorprinzi and colleagues provide insights into the effects of exercise-induced arousal on memory storage and retrieval. Frith et al. (2017) were among the first to examine in a single experiment the impact of the temporal pairing of acute exercise and encoding on memory. Young adults were assigned to separate conditions in which they performed a bout of exercise either prior, during, or following encoding or a no-exercise control condition. Exercise consisted of a 15-min treadmill run in which the first 5 min was jogging (RPE 11–12), the next 5 min was spent running at faster pace (RPE 13–15), and the final 5 min was spent running at a hard pace (RPE 16–20). Pacing at each stage was self-selected. During encoding periods, participants listened to a list of 15 individually presented words which were presented successively 5 times (Rey Auditory Verbal Learning Test- RAVLT). Free recall memory was assessed 20-min post encoding and a recognition test of memory was taken 24-h post encoding. The recognition test consisted of the presentation of the 50 words heard during encoding and 20 new distractor words. Performance was measured in terms of recognition and list attribution. Prospective memory was quantified by a time-based procedure in which participants were asked to telephone the researcher at an agreed upon time. When measured after a 20-min delay, participants in the simultaneous encoding group and the control group recalled significantly fewer words than participants who exercised prior to encoding. There were no differences in participants’ free recall regardless of condition when evaluated after a 24-h delay. Word recognition differed among the groups; individuals in the simultaneous encoding and post-encoding conditions performed significantly poorer than those who exercised prior to encoding. Measures of prospective memory did not differ among groups.

In a similarly designed study, Sng et al. (2018) compared long-term memory and prospective memory in groups of young adults who either rested or performed a low intensity 15-min treadmill walk prior to, during, or following encoding. Participants completed the RAVLT during encoding periods. Free recall memory was assessed 20-min post encoding and a recognition test of memory was taken 24-h post encoding. Prospective memory was measured by the Red Pen Test, which provided an index of participants’ ability to remember to execute a specific response. Measured at both 20-min and 24-h delay periods, participants who simultaneously walked and encoded the word list performed significantly poorer than those who exercised before encoding. Notably, there was no significant difference between those who exercised prior to encoding and the control group. In addition, the prospective memory of participants in the simultaneous condition was significantly poorer than that of participants in either the before condition or the control condition. The lower memory performance of participants in the simultaneous condition compared to the before exercise condition was explained by Sng and colleagues as the result of competition for attentional resources (Dietrich and Audiffren, 2011).

Tomporowski et al. (2017) investigated the effects of physiological arousal produced by isometric muscle contractions on free-recall and recognition memory. Using a within-participant and counterbalanced design, young men, and women studied different 20-item word lists under four conditions: a 100-s hand-grip contraction during word encoding, consolidation, retrieval, and a no-contraction control condition. Arousal was manipulated by assigning participants to either a low-intensity (10% maximum grip strength contraction) or a moderate-intensity (50% maximum contraction) exercise group. A free-recall test was administered immediately following each trial and a comprehensive delayed free-recall test was given 5-min following the last trial. A recognition memory test was administered immediately following the delayed free-recall test. One hundred words were visually presented; 50 of the words had been presented during the test and 50 words had not been presented. The participant was asked to indicate whether an item was from one of the word lists presented earlier or whether it was a new word. Isometric exercise was not performed during either the comprehensive recall test or the recognition memory test. Statistical analyses revealed that arousal level did not differentially influence encoding. However, fewer words were recalled during immediate and delayed free-recall tests and recognition tests under conditions in which participants performed the handgrip exercise while encoding than when exercise was performed during recall, consolidation, or control conditions. The researchers surmised that dual-task costs are particularly high during the encoding phase of learning.

#### Summary of Arousal Theory

All studies reviewed here on the basis of hypotheses drawn from arousal theory and accompanying neuro-psychological based models consistently show evidence that a combination of exercise and encoding negatively impacts long-term declarative memory. Several experiments make clear comparisons between conditions in which exercise is performed concurrently with encoding and conditions in which exercise is performed prior to or following encoding. The evidence in these studies depicts less efficient long-term memory storage of declarative information under the former condition. Further, there is little support for an inverted-U relation between exercise intensity and memory of declarative information.

## Attention Theory

The relation between attention and memory has been studied extensively for over a century (see Mulligan, 2008 for a review). Attention is typically viewed as a focusing process that plays a critical role in encoding, short-term memory, and long-term memory (see Jonides et al., 2008, for a review). The focus of attention can be placed on encoding, which is a perceptual process, as well as memories stored from past experiences. Information that is encoded and comes into the focus of attention can displace other memory contents from attentional focus. Attentional processes control what information enters into the focused state. This on-line processing explains the limited storage capacity of short-term memory. Attentional processes not only serve as “gating” mechanisms and determine what enters consciousness, but also how the information is maintained, and how additional information is retrieved from short-term and long-term memory storage. Memory performance degradation is typically explained in terms of either the decay of memory traces (engrams) in cortical structures (Loprinzi et al., 2017) or the interference among items held in perceptual memory, short-term, and long-term memory (Jonides et al., 2005). Of central importance to the present review is how concurrent physical activity may moderate the ability to overcome interference.

### Attention Research: Experimental Evidence

A series of experiments examined the effects of walking on young and older adults’ memory. The studies were designed to test predictions derived from the Selection, Optimization, and Compensation (SOC) theory (Baltes and Baltes, 1990; Baltes and Lindenberger, 1997), which focuses on age-related shifts in the quality of sensorimotor processing. The SOC theory posits that individuals respond to environmental challenges via selection and modification of task goals and optimization of goal-directed compensatory strategies required to achieve those goals. With age-related declines in sensory acuity and proprioception, older adults are predicted to allocate increasingly more attentional resources to maintaining desirable levels of balance and walking control. Lindenberger et al. (2000) employed a dual-task methodology to assess the magnitude of dual-task interference between memorization of words and walking. 47 young (ages 20–30 years), 45 middle-aged (40–50), and 48 old (60–70) adults were trained to walk quickly and accurately on narrow paths that differed in movement complexity. In separate sessions, participants encoded 16 words presented auditorily while sitting, standing, or walking for 2.8 min on either track. Immediate serial recall tests were administered. Recall performance in the context of walking was lower than recall performance in the context of seated and standing conditions. Middle- and older-age participants showed a 22% loss in serial recall under a simple walk condition and a 36% loss during the complex walk whereas young adults showed no loss following the simple walk and a 19% loss during the complex walk. Similar findings were obtained from a subsequent study (Li et al., 2001) which retained the methods employed by Lindenberger et al. (2000) but individualized the walking and memory demands for each participant. Compared to a seated condition, older adults (60–75 year) showed significantly poorer serial recall performance following walking than did young adults (20–30 year). Younger adults showed no dual-task interference effect when performing a simple walk condition, and interference only when the walking task was more challenging (e.g., path obstacles).

A series of experiments conducted by Helton and colleagues focused on dual-task challenges presented in naturalistic conditions. In one study (Epling et al., 2016), young adults were asked to remember 20 words presented auditorily at 15-s intervals during a vigorous 5-min run on an outdoor running track. Immediately following the run, participants completed a 90-s written word-recall test. Significantly fewer words were recalled following the run than when the participant encoded words while seated. Similar results were obtained in two studies that evaluated dual-task interference when encoding words while performing traverse wall climbing (bouldering). Green and Helton (2011) assessed dual-task costs of bouldering across a climbing wall for three minutes and encoding 20 words presented auditorily every 8 s with a 14-s pause after the final word. Immediately following the climb, participants completed a 90-s written word-recall test. Compared to a single-task condition in which encoding occurred while seated, the dual-task condition resulted in a 50% decrease in recall performance. In a replication study, Darling and Helton (2014) presented words with irregular timing and the duration of traverse climbing was increased to 5 min. Compared to word recall performance in a single non-exercise condition, participants recalled nearly 40% fewer words. Results of these two studies suggested to the researchers that effortful processing required to traverse the wall climb interfered with the rehearsal and maintenance of words to be recalled.

#### Summary of Attentional Allocation Research

All studies reviewed here that were conducted on the basis of hypotheses drawn from attention theories consistently show evidence of CMI that negatively impacted the encoding of declarative information into long-term memory. The magnitude of interference appears to be age-related, with middle-age, and older adults showing less effective word memorization than younger adults. The data suggest that dual-task conditions that require motor movement planning and corrections compete with the ability to retrieve strategies from memory storage that are required for processing cognitive tasks, and thus negatively affect the encoding of semantic information into long-term memory storage.

## Cognitive-Energetic Theory

Proponents of cognitive-energetic theories suggest that traditional information-processing models that view processing systems as operating in a computer-like, mechanical, “dry” fashion are insufficient because they fail to acknowledge the importance of “wet” biological and motivational systems that underlie top-down control of behavioral output (Koelega, 1996). These theories draw from long held assumptions concerning an interrelation between top-down cognitive control and bottom up sensory input and processing (Kahneman, 1973; James, 1981/1890; Hockey, 1996, 1997; Sanders, 1997; Audiffren, 2009). Central to the early theoretical conceptualization of attention was the assumption that it guides behavior via the allocation of mental resources. Further, the amount of attentional resources available for an individual was believed to be fixed. As a consequence, attentional capacity is allocated in ways that optimize behavioral actions and their consequences when an individual is faced with performing two or more tasks at the same time (Kahneman and Treisman, 1984). While there is theoretical debate concerning whether a single central reservoir of attention or multiple reservoirs exists (Wickens, 1984, 2008), predictions concerning dual-task demands on performance are similar – when two or more tasks compete for available attentional resources, the resulting condition has the potential to lead to decrements in performance in one task in favor of the other(s).

While several contemporary cognitive-energetic theories address factors that explain dual-task performance (Dietrich and Audiffren, 2011), Cognitive Load theory (Paas and Sweller, 2012) is particularly relevant to the present review. The theory makes specific hypotheses concerning the bidirectional relation between working memory and long-term memory stores. The theory posits that the limited operational capacity of working memory is offset by the availability of schemas stored in long-term memory. Further, the primary role of working memory is to assess on-line processing of sensory experiences and to determine if they are unique, novel, and merit encoding. As schemas are hypothesized to consist of multiple elements of information that are reduced into a single element, they provide the means to overcome the computational limitations of working memory. Drawing on an evolutionary theory of human cognitive architecture (Geary, 2006), Paas and Ayres (2014) suggest that schemas that control motor movements take precedence over schemas that organize and refine the acquisition of cultural knowledge (e.g., semantic information). Paas and Sweller (2012) suggest that primary motor schemas can be used to leverage the encoding of secondary, academic material. Environmental and instructional conditions that reduce cognitive load on working memory are hypothesized to facilitate encoding and learning (Choi and van Merrienboer, 2014).

### Cognitive Load Theory: Empirical Evidence

Educationally-oriented researchers have particular interests in interventions that can enhance children’s memory and learning. A recent series of studies conducted with children addressed the impact of dual-task instruction on long-term memory. A study conducted by Toumpaniari et al. (2015) provided the rationale and methodology employed in several experiments. Pre-school boys and girls were taught a foreign language vocabulary via the pairing of Greek and English animal words. When presented an animal word, groups of children either physically moved and imitated animals (integrated movement), remained seated and gestured animal movements, or remained seated and verbally repeated the words. Twenty word pairings were practiced 1 h per day, 2 sessions per week for 4 weeks. The results of an immediate cued-recall test administered following the last encoding session revealed that children who were physically active and imitated animal actions recalled significantly more words than children in the gesture-only condition. Children in both physical activity and gesture groups remembered more words than children in the traditional verbal-repetition group. These results were replicated in a cluster randomized control experiment conducted with 104 elementary-age children (Schmidt et al., 2019). Groups of children between 8 to 10 years of age were instructed to associate 20 foreign animal words with 20 known animal words during four, 10-min teaching sessions that were distributed over 2 weeks. Word pairs were presented auditorily and pictorially to children who repeated the word pairs while either enacting the movements of the animal, running in place, or seated a desk. A cued-recall test administered following the final training session revealed that word recall was greater for children who were physically active during encoding than for children who were inactive. Further, the greatest memory gains were shown by children who enacted animals’ actions.

These findings were supported by a series of experiments that focused on words acquired during geography, science, and mathematics instruction. In the first study Mavilidi et al. (2016), children ranging between 4 and 5 years of age participated in classroom lessons designed to associate animals’ names with specific global continents. A large map of the globe was placed on the floor. Children paired an animal’s name to a location on the map in one of three ways: physical movement that imitated the animals’ “travel” to a geographical location (integrated condition); picking up an animal doll and running in a circle around the map (physical activity condition); or visually locating the animal’s place on the map (control). Encoding training was conducted in three, 10-min periods over 2 days. Cued recall tests were administered immediately following the second learning day and again after 5 weeks. Children who learned under the integrated condition and the physical activity condition recalled more words than children under the control condition on both immediate and delayed memory tests. Word recall in the integrated learning and the physical activity learning groups did not differ. Similar results were obtained in cluster randomized experiment in which preschool children participated in a science lesson that involved learning the names and positions of planets in the solar system (Mavilidi et al., 2017). The sun and drawings of named planets were displayed in order on a straight line in the solar system task. During 10-min sessions conducted once per week for 4 weeks, children in the integrated instructional group ran from the center (the sun) to a planet indicated by the teacher and then back to the center, continuing for each planet. Children in the run condition were instructed to run around the solar system for several minutes and then sit and listen to the teacher name the planets. Children in the control group remained seated and listened to the teacher. Instruction was provided once each week for 4 weeks. Free-recall and cued-recall tests were administered immediately after the final practice session and again after 6 weeks. An analysis of scores combined from the two test sessions revealed that children in the integrated condition group performed better than those children in the nonintegrated (run) and control condition groups. Further, children in the “run” condition group performed better than those children in the control condition group. Another cluster randomized control trial conducted with preschool age children (Mavilidi et al., 2018a) focused on numeracy skills. One hundred and twenty preschool students received practiced counting skills during four weekly 15-min sessions. Children were assigned to one of four conditions: an integrated physical activity in which they ran, jumped, and stepped along a number line while counting; a non-relevant physical activity in which they ran around the room for 1 min; a condition during which an observer watched the students perform the integrated movements, or a seated control condition. Children’s numeracy skills were assessed immediately after training and 6 weeks following the intervention. Children in the integrated, task-relevant performed significantly better than children in all other conditions.

#### Cognitive-Energetics Theories: Summary

Experiments that integrate children’s gross motor movements into problem solving tasks performed in classroom setting consistently report improvements in the encoding of semantic information. Unlike laboratory studies, children engaged in learning sessions distributed across several days. Further, instructional environments were purposely designed to link physical activity to the creation of mental representations (e.g., schemas). As predicted by proponents of cognitive load theory, physical movements may provide leverage for encoding and remembering academic information. Planned physical actions result in sensorimotor feedback that is processed and embellished via memories of past experiences and enhance memory trace strength.

## Entrainment and Embodied Learning Theories

Physical activity leads to widespread biological changes that are rhythmical in nature (e.g., cardiorespiratory rate, relative timing of muscle activation, and brain activity). The importance of genetically ingrained motor movement programs and their role in encoding and storage of semantic information is central to proponents of entrainment theory. Entrainment theory builds on the dynamic nature of human movement and the temporal locking between an individual’s motions with the frequency of another external rhythm (Thaut et al., 2005; Thaut et al., 2015). The phenomenon is exemplified by the linkage between the auditory rhythms embedded in music and the regulation of spatiotemporal and force parameters of movement (e.g., sway or groove). Proponents of Dynamic Attending Theory (Jones and Boltz, 1989; Large and Jones, 1999) hypothesize that the temporal structure of movements provides an endogenous template for information encoding. Rhythmic sensory inputs during movement phases are theorized to be linked to the peaks of oscillating attentional phases. The timing of specific motor movement phases (e.g., walking, running, or cycling) and accompanying peaks of oscillatory attention provides optimal conditions for encoding. Thus, learning would be predicted to be facilitated when the presentation of information to be learned (e.g., word list items) occurs in phase with periods of peak attention. Alternatively, as the out-of-phase timing between movement and encoding increases, learning is compromised. These predictions are supported by recent neurophysiological studies that have mapped direct paths and indirect paths of connectivity between networks that comprise executive functions and the hippocampi (Eichenbaum, 2017). These predictions dovetail with those of embodied learning theories proposing that learning emerges from a dynamical interaction among an individual’s body movements, the sensory experiences obtained from the movements, and the context of those movements (Newell, 1986; Lindgren and Johnson-Glenberg, 2013). As individuals navigate through complex environments and perform actions, a framework is developed that allows them to learn about the world and how actions provide the means to achieve goals. Memory of movements is thought to be retained in real time and depends on the sensory and motor experiences obtained during physical actions. Learning is hypothesized to be grounded in enactment, in which memories are encoding through sensory experiences derived from such physical actions as gestures, walking, and play (Lindgren and Johnson-Glenberg, 2013; Gallagher and Lindgren, 2015). The movements that occur during enactment are hypothesized to engage not only the motor system but also to facilitate the construction of mental representations that enhance memory recall (Moreau and Tomporowski, 2018).

### Entrainment Research: Experimental Evidence

A series of studies conducted by Schmidt-Kassow and colleagues provide support for the beneficial role of entrainment on memory storage. Young women in one experiment participated in two identical laboratory sessions in which separate groups of participants rested or cycled on an ergometer at a low-to-moderate intensity for 30 min either prior to or during encoding auditorily presented word lists (Schmidt-Kassow et al., 2013). Individual words were presented every 2 and 6 s and corresponded to the 60-RPM cycling cadence maintained by the participant. This manipulation was based on prior research demonstrating the temporal predictability benefits of acoustic stimuli (Schmidt-Kassow et al., 2010; Schmidt-Kassow et al., 2017). Cued-recall tests were administered 48 h following encoding. Significantly more words were recalled in both sessions when encoding occurred simultaneously with cycling exercise than when a rest period preceded encoding. Word recall performance of participants who exercised prior to encoding did not differ from that of participants in either the simultaneous or rest conditions.

A subsequent study provided additional support for the benefits of pairing acute physical activity with encoding (Schmidt-Kassow et al., 2014). In two separate experiments, young men, and women completed two sessions during which they either encoded a list of words while treadmill walking or remained seated. Each participant was provided treadmill familiarization training and his or her preferred walking speed was identified. The participants’ walking speed was synchronized to the presentation of individual words. A paired-association paradigm was employed in which 40 Polish-German words were paired. One word of each pair was presented every fourth step. Participants verbally repeated the word pairs during an 8.2 s (12-step) separation between word pairs. The word-pair list was presented twice during the 30-min treadmill walk. In both experiments, cued-recall tests were administered 24 h after each session. The only methodological difference between the experiments was blood draws that yielded separate evaluations of the kinetics of brain-derived neurotropic factor (BDNF) and cortisol. The behavioral data were consistent across both experiments, with superior word recall occurring following concurrent exercise-encoding sessions compared to non-exercise conditions. The research conducted by Schmidt-Kassow and colleagues highlight the fact that subtle differences in the synchronization of movement with word presentation can exert substantial effects on long-term memory.

#### Summary of Entrainment Research

While limited in number, the studies designed on the basis of hypotheses drawn from entrainment theory consistently provide evidence of long-term memory facilitation. As predicted, gains in long-term memory were observed following the coupling of locomotor movements with word presentation. Central to the methods employed in these studies is the precise timing of the presentation of words to be remembered with cycling cadence and with self-paced walking. As predicted by proponents of entrainment theory and embodied learning theory, memory storage may reflect the results of a dynamic interplay among environmental information, task constraints, and organism constraints. Central to embodied learning theory is an assumption that muscle coordination, control, and skill emerge as self-organizing coordinative structures through the unity of perception and action.

## Discussion

The pairing of physical activity with cognitive task performance constitutes a dual-task condition and the potential for CMI. The manner in which interference affects cognitive performance or movement performance depends on several factors. The goal of the review was to use contemporary cognitive theory to search for consistent themes that explain outcomes from learning conditions that combine motor movements with the encoding of semantic information. The rationale for the review was based on evidence showing the multicomponent training conditions that pair physical activity with mentally engaging tasks result in higher gains in cognitive performance than when physical activity is performed in isolation (Tomporowski and Pesce, 2019). Further, educators, mental health practitioners, and gerontologists have considered the merits of physical activity interventions designed to enhance or maintain cognitive functions. As such, we were particularly interested in dual-task studies that lead to long-term improvements in learning.

While relatively few experiments have been conducted that study the phenomenon, the results of the 16 studies evaluated here provide some resolution concerning specific dual-task conditions and their outcomes. As predicted by the CMI task classification system developed by Plummer et al. (2013), some of the studies led to impaired memory storage while others provided evidence of memory enhancement. Dual-task studies resulting in degraded long-term memory performance measured semantic memory following encoding that occurred while walking on pre-arranged pathways that included obstacles (Lindenberger et al., 2000; Li et al., 2001), engaging in speeded traverse wall climbing (Green and Helton, 2011; Darling and Helton, 2014), maintaining specific levels of muscular exertion (Tomporowski et al., 2017), and cycling or running at paced speeds (Epling et al., 2016). Studies finding improvements in long-term semantic memory measured young children’s learning in immersive classroom activities that involved running while acquiring vocabulary words (Toumpaniari et al., 2015; Schmidt et al., 2019), animal names (Mavilidi et al., 2016), planet names (Mavilidi et al., 2017), and numeracy (Mavilidi et al., 2018a). Similarly, young adults’ memory of words was found to be greater when words were presented in synchrony with ergometer cycling pacing (Schmidt-Kassow et al., 2013) and self-paced treadmill walking (Schmidt-Kassow et al., 2014).

Contemporary theoretical views of arousal, attention, working memory, and memory storage are central to explaining the differences among these studies. Attentional focus is thought to determine the entry of information into short-term working memory where the capacity limitations of short-term memory are considered crucial for establishing long-term memories (Jonides et al., 2008; Paas and Ayres, 2014). Executive functions are central to the planning, selection, and guidance of movements (Diamond, 2013). Further, the proprioceptive feedback that occurs during movements and evaluation of the consequences of actions are interpreted by executive functions (Schmidt, 1975). Based on capacity theories of attention, it would be expected that the processing required to control motor movements and update sensory feedback would compete for available computational resources. From these theoretical perspectives, declines in semantic encoding would be predicted.

Explaining improvements in semantic memory storage under dual-task conditions that include motor movement presents a challenge for traditional attention theories. One method of identifying factors that contribute to improved learning under dual-task conditions is to rule out possible explanations. For instance, experiments that examine the role of automaticity on dual-task costs (Schneider and Shiffrin, 1977; Shiffrin and Schneider, 1977) report results suggesting that protracted practice on one task leads to less top-down processing and reduction in working memory requirements. However, none of the studies reviewed here reporting memory enhancement included extensive motor-movement training. Alternatively, data obtained from memory research suggests that emotionally laden events that evoke intense physiological stress responses provide the basis for flashbulb memories, which are vivid and may be stored virtually indefinitely in episodic long-term memory (Hirst and Phelps, 2016). Episodic memories are considered to be a type of declarative memory that is unique to an individual and reflects personal experiences (Squire and Wixted, 2011). Many individuals can recall in great detail experiences encoded during intense sport training and competition. Such observations have led researchers to conclude that high-intensity exercise may provide conditions that enhance the encoding of events that occur prior to, during, and following intense, stressful bouts of physical activity (Lucas et al., 2015). As described previously, research conducted on stressors that elicit fear patterns in animals (e.g., startled response and freezing) heighten episodic and spatial learning (McGaugh, 2018). These changes in learning are explained in terms of limbic-based structures and networks that involve the amygdala and hippocampus and signal widespread homeostatic responses and regulation. However, none of the dual-task experiments evaluated in the present review employed the levels of intense physical activity that might lead to stress levels that elicit strong emotional responses. Further, a recent meta-analysis found little difference between the effects of acute bouts of moderate or intense exercise on cognitive function (Moreau and Chou, 2019).

The dual-task studies that led to improved semantic memory are characterized by game-like play, self-paced movement, and the manner by which information to be learned was timed with physical movement. The finding that game-like academic learning that involves the pairing of physical activity with encoding of words is in line with positions held by researchers who have proposed the importance of the qualitative aspects of chronic physical activity interventions. The value of physical activities that are meaningful, goal-directed, and pleasurable are hypothesized to enhance execute functions (Diamond and Ling, 2016; Tomporowski and Pesce, 2019). The beneficial effects of game-like activities on children’s memory also support predictions concerning the interactive role of cool and hot executive networks (Zelazo and Carlson, 2012). The affective responses individuals derive from physical movements while immersed in goal-directed learning may alter motivation and level of engagement (Stych and Parfitt, 2011; Vazou and Smiley-Oyen, 2014; Vazou and Skrade, 2017). The neural connectivity between structures of the pre-frontal cortex and the hippocampus has been described recently and may help explain how low-to-moderate levels of mentally engaging exercise paired with goal-directed behavior might provide the basis for enhanced memory storage (Eichenbaum, 2017).

Neurobiological research provides evidence of direct and indirect connections between the prefrontal cortex and the hippocampus and that memory storage is mediated by oscillatory synchrony of neural activity (Eichenbaum, 2017). The finding that the timing between movements and memory encoding enhances memory supports predictions made by Dynamic Attending theory (Large and Jones, 1999), which emphasizes the role of the timing of biological rhythms inherent in movement with sensory experiences derived from motor actions. Self-paced treadmill walking has been shown to improve children’s (Schaefer et al., 2010) and older adults’ (Tomporowski and Audiffren, 2014) executive processing. On the basis of entrainment theory, participants in these studies may have adjusted their walking pace to synchronize movements with the timing of information entering working memory. Treadmill walking and highly practiced motor skills are often assumed to be highly reflexive and require limited attentional control (Regnaux et al., 2006). However, attempts to alter or modify ingrained actions require effortful attention processing that competes for mental resources that could be allocated to other tasks (Beilock et al., 2002). It is plausible that dual task conditions that minimize top-down motor movement control lead to improvements in declarative learning by providing additional working memory space.

## Conclusion

The results obtained from 16 experiments suggest that declarative memory can be enhanced under specific dual-task conditions. However, several caveats are in order. The experiments selected for review were not derived from a systematic literature search. The studies were linked to four overlapping categories of theories: arousal, attention, cognitive-energetic, and entrainment theory. Our theory-based approach was designed specifically to target dual-task experiments that examined memory and learning outcomes. The intent was to identify conditions that create cognition-motor interference but benefit declarative memory. As predicted, the differences observed in the outcomes of studies reviewed are related to subtle methodological factors. For example, the relation between exercise and memory may be due to the timing of memory testing. Roig et al. (2016) highlighted the importance of delayed tests of long-term memory. Memory testing conducted immediately or soon after encoding in their research provided negligible learning. However, testing conducted 24 h and 7 days following encoding provided unambiguous evidence of the effects of acute exercise on procedural learning. It is noteworthy that 8 of the 16 studies reviewed included memory tests administered 24 h or longer after the learning phase and that 6 of the 8 experiments support the provision of sufficient time for the consolidation of long-term memory.

Further, there is a general consensus that there are several different types of memory; e.g., perceptual, procedural, episodic, semantic, and spatial (Schneider, 2015). Advances in technology and neuroscience have led to a better understanding of brain structures and functions that help explain how human experiences lead to the organization and re-organization of knowledge. While considerable advances in understanding the linkages between physical activity and memory have been made recently, attempts to explain the relationship will benefit from a wider selection of tests of memory than those currently populating the literature. Soga et al.’s (2017) experiment exemplifies the strength of evaluating multiple types of memory and identifying dissociations in outcomes that conform to theoretical predictions. At a more elementary level, it will also be informative to examine closely the information-processing characteristic of memory tasks selected for experimentation. Many of the 16 studies reviewed that failed to observe memory benefits employed tests that presented to-be-remembered items at a relatively fast rate. Most of the experiments that observed beneficial effects either paced the presentation of words with movements (e.g., Schmidt-Kassow et al., 2014) or provided considerable delays between items to be remembered (e.g., Toumpaniari et al., 2015; Mavilidi et al., 2017). Additional research focused on the timing of motor movement and rate of presentation of information to be encoded may address theoretical questions concerning the role of central (e.g., Kahneman, 1973) or distributed (e.g., Wickens, 1991) attentional resources, as well as the clinical applications of dual-tasking interventions. Regardless, research that looks closely at the type and the characteristics of memory tasks will be useful for theory development and subsequent applications to multiple populations.

Developmental and aging factors are also of particular importance. Five studies performed in educational settings demonstrated children’s retention of academic information. However, children in these experiments engaged in several dual-task training sessions that were distributed over several weeks. These studies differ from traditional acute dual-task training experiments, which are limited to assessing the effects of a single exercise bout. While they might be considered to be chronic exercise interventions, the total memory encoding durations were similar to those of single session experiments. Additional studies that track the strength of memory encoding over multiple training sessions are needed. It is unknown if the game-like conditions experienced by children have similar effects on adolescents’ and adults’ learning.

There is considerable interest in determining whether school lessons that include physical activity will boost children’s academic progress (Vazou and Smiley-Oyen, 2014; Donnelly et al., 2016; Daly-Smith et al., 2018; Mavilidi et al., 2018b) and whether multi-component activities help older adults offset or reverse age-related changes in cognition (Tomporowski and Pesce, 2019). Clearly, additional theory-based research is needed that provides guidance concerning the application of physical activity interventions designed to enhance cognition. Theory-based literature reviews geared toward explaining the relationship between exercise and cognition provided added value to traditional meta-analytic reviews that describe and quantify the strength of the relations that exist between exercise and cognition.

## Author Contributions

PT performed the literature search. PT and AQ drafted the manuscript, and both authors approved the final version of the manuscript.

## Conflict of Interest

The authors declare that the research was conducted in the absence of any commercial or financial relationships that could be construed as a potential conflict of interest.
